# The Usefulness of a Wearable Electronic Vision Enhancement System for People With Age-Related Macular Degeneration: A Randomized Crossover Trial

**DOI:** 10.1167/tvst.14.9.8

**Published:** 2025-09-04

**Authors:** Andrew Miller, Michael D. Crossland, Jane Macnaughton, Keziah Latham

**Affiliations:** 1 Vision and Hearing Sciences Research Centre, Anglia Ruskin University, Cambridge, UK; 2 College of Health and Life Sciences, Aston University, Birmingham, UK; 3 UCL Institute of Ophthalmology, London, UK; 4 NIHR Moorfields Biomedical Research Centre, London, UK

**Keywords:** AMD, Head-mounted display, image enhancement, low vision aid, visually impaired persons, wearable devices, wEVES

## Abstract

**Purpose:**

To determine the usefulness of a wearable electronic vision enhancement system (wEVES) for people with age-related macular degeneration (AMD).

**Methods:**

Thirty-four adults with AMD, 64.7% female, mean age 80.2(±6.0), were recruited from a UK low vision service. A 12-week non-masked randomized crossover trial compared wEVES usefulness with participants’ existing low vision solutions. Primary outcome measures were visual ability, vision-related quality of life (VRQoL), device usage, and user-reported preferred device. Secondary outcomes were adverse effects, willingness to purchase, and qualitative reactions.

**Results:**

Overall visual ability improved with wEVES compared to existing solutions alone (mean difference −0.26; 95% confidence interval [CI], −0.48 to −0.04; *P* = 0.02). The wEVES were used for varied activities, including distance tasks, with few reported alternative strategies. However, these findings did not translate into changes in VRQoL (mean difference 0.10; 95% CI, −0.27 to 0.46; *P* = 0.59) or sustained device use. The wEVES were not the most preferred device for any task or individual, even when self-reported performance surpassed existing solutions. Adverse effects were minor, but participants’ satisfaction and willingness to use wEVES declined significantly from trial baseline to end.

**Conclusions:**

The wEVES improved self-reported visual ability, indicating their potential to support vision rehabilitation for people with AMD, albeit in a device that was largely not preferred over existing solutions. A user-led home trial evaluated using mixed methods is more indicative of the usefulness of wEVES for people with AMD than a short clinical demonstration.

**Translational Relevance:**

To understand the usefulness of wEVES for people with AMD, broader measures than visual function and visual ability should be applied within longer user-led assessments.

## Introduction

Age-related macular degeneration (AMD) causes loss of central retinal function and is associated with declining visual acuity, functional ability and quality of life.^1^^–^^4^ In 2020, global estimates suggest that AMD was the cause of blindness for 1.85 million people and moderate to severe vision impairment (VI) for another 6.23 million,^5^ with total prevalence estimated to reach 288 million by 2040.^6^ Treatment options for both wet and dry forms of AMD continue to emerge, but many people are left with significant levels of VI and require rehabilitation assistance.^7^ Magnification, lighting advice, and contrast enhancement are the principal modifications used to offer support,^8^ with mainstream electronic devices and specialist assistive technology (AT) increasingly being used by people with all forms of VI,^8^^–^^10^ to improve their health outcomes.^11^

Wearable electronic vision enhancement systems (wEVES) were first developed in the early 1990s^12^^,^^13^ and are an example of AT designed specifically for people with VI. wEVES are head-mounted devices with screens in front of each eye displaying real-time images from a worn camera. The displays can show images from near, intermediate, and far distances, allowing the user to manipulate the levels of magnification, contrast and brightness. Despite limited evidence, wEVES are frequently marketed as suitable AT for people with AMD and can be broadly split into two categories^14^:

1. Enclosed devices provide complete visual immersion, with brighter and broader images than alternatives. Examples include IrisVision Live 2.0 (irisvision.com/irisvision-live-2-0) and GiveVision (givevision.net/en/sightplus).
2. Non-enclosed devices are lighter, more spectacle-like devices that allow users to see their surroundings around the screen content. Examples include eSight Go (esighteyewear.com/esight-go) and NuEyes Pro 4 (www.nueyes.com/pro4).

In mixed populations of people with differing causes of sight loss, wEVES have been shown to improve measures of visual function,^14^^–^^20^ vision-related quality of life (VRQoL)^16^^,^^21^ and some instrumental activities of daily living.^14^^,^^18^^,^^22^^–^^24^

AMD is a chronic disease affecting older adults, with prevalence increasing most rapidly after age 75.^6^ It is known that older adults are slower adopters of new AT, using a more limited range of functionality, which may be due to experiential, physiological, and cognitive factors,^25^ as well as inaccessible design,^26^ slower training uptake,^27^^,^^28^ and a lack of suitable training resources.^29^^,^^30^ Therefore it is important to evaluate the views and needs of people with AMD separately from those of the wider VI population. Additionally, much of the earlier research compares wEVES with baseline measures, which helps demonstrate utility but is of more limited interest to practitioners and users wishing to understand whether this emerging form of AT provides benefits compared to participants’ existing low vision solutions.^31^

International standards recommend using randomized control trials (RCT) to evaluate the medical uses of virtual reality and augmented reality products.^32^ Earlier work investigating wEVES are primarily observational studies in mixed populations with no control group,^31^ creating a recognized unmet need for an RCT to evaluate the usefulness of wEVES.^33^^,^^34^ Competitive enablement^35^^,^^36^ is a user-centered conceptual approach that assesses the suitability of assistive devices alongside alternative solutions in user-identified problematic tasks. Assessing AT's usefulness within tasks of “high functional relevance” to the participants has been proven to lessen abandonment rates^36^ and, by extension, can improve the relevance of findings to the targeted population. This conceptual approach was chosen to evaluate desktop CCTV magnifiers for people with AMD,^37^ because it allows participants to explore the relative rather than absolute benefits of a device. The invitation to compare a new solution with existing ones requires participants to judge success on a broader range of factors than the device's functional delivery alone, producing findings that may be more directly relevant to potential consumers and practitioners. An RCT for people with AMD was designed with the following hypotheses: Compared with existing low vision solutions, wEVES will:

(1) Reduce self-reported activity limitation.
(2) Be more versatile, allowing use for tasks without a current solution.
(3) Be the preferred low vision aid (LVA) and be used more often than existing solutions.
(4) Improve VRQoL.
(5) Be a cost-effective solution in improving the QoL for people with AMD.

Evidence regarding the benefits of wEVES will support the prescribing decisions of practitioners exploring novel solutions and the corresponding purchasing decisions of people with AMD. In addition, it is imperative that manufacturers are informed by independent, patient-centered research findings when developing future iterations of wEVES.

## Methods

### Participants

Participants were recruited by convenience and snowball sampling from a low vision service in Birmingham, UK and local Macular Society support groups. Participants with VI, primarily due to AMD, were invited to take part, with inclusion and exclusion criteria shown in Table 1.

**Table 1.** Inclusion and Exclusion Criteria for the Trial


Inclusion criteria
Vision loss is primarily due to AMD, which affects the participant's daily life
People who are 18 years or over
Able to undertake assessments in English
Passed the short form Mini-mental State Evaluation (adapted for vision loss)^38^
Able to operate the controls of the wEVES
Not an existing wEVES user
Hearing sufficient to undertake a telephone interview
Exclusion criteria
Existing wEVES user
Other forms of ocular pathology causing significant vision impairment
Significant deterioration in vision reported in the two months before entry
Planned curative ocular surgery (e.g., cataract operation) in the study time
Balance-related disorders (e.g., Ménière's disease)
Heart disease
Uncontrolled high blood pressure
Visual acuity is good enough to hold a current UK driving license (e.g., 6/12 [20/40] or better without field loss)

### Device Used in the Trial

**Figure 1.**
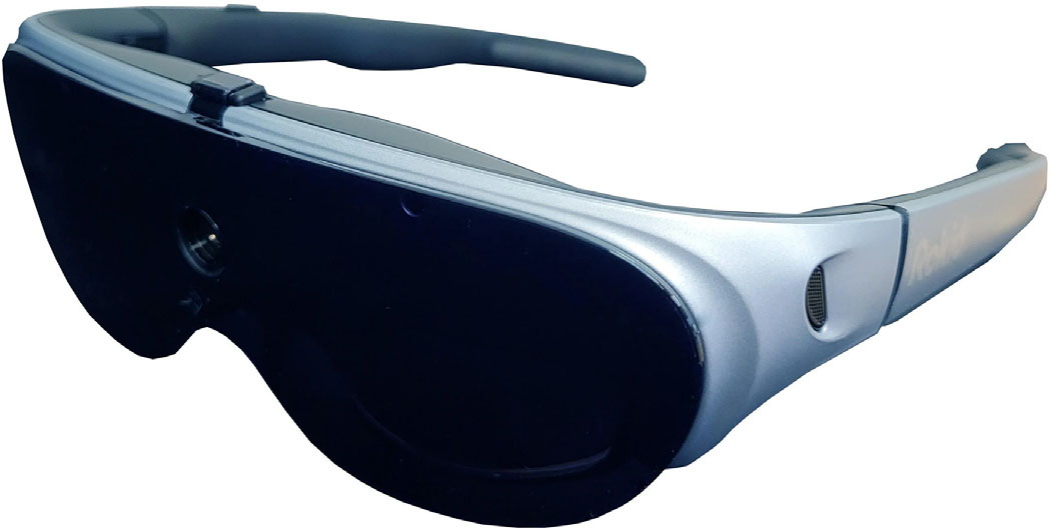
Eyedaptic Eye5 Headset (Image with permission https://www.visionaid.co.uk/wearable-solutions/eye5).

**Figure 2.**
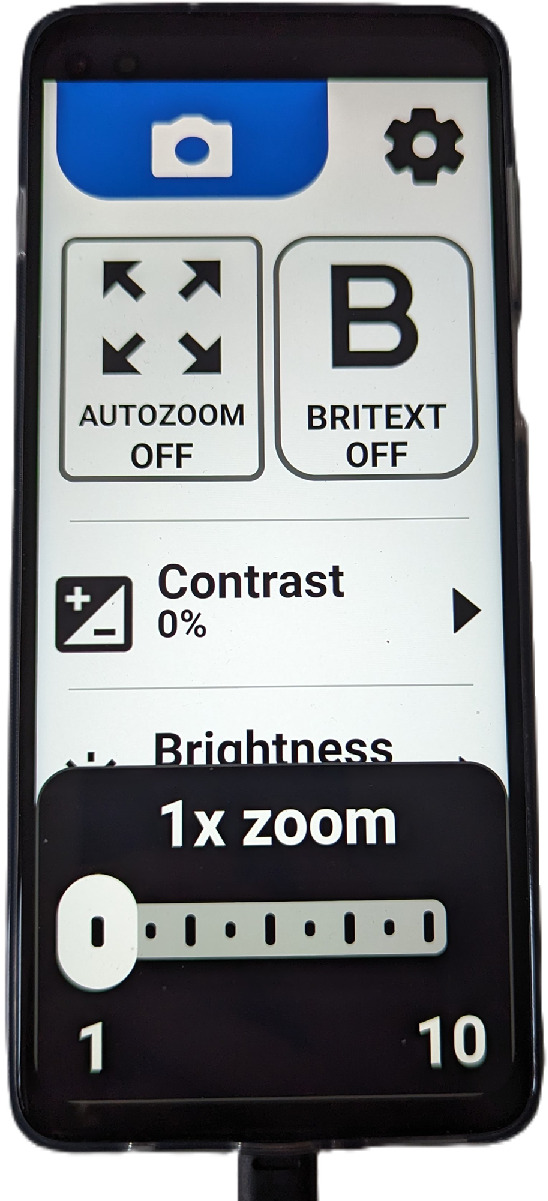
Eyedaptic Eye5 Tethered handset with controls (image Author's Own).

A patient involvement and engagement event was held to select the device to be used from a representative sample of wEVES currently available in the UK. The device chosen was the Eye5 (Eyedaptic California USA), which comprises a non-enclosed spectacle-style headset tethered by a wire to controls on a repurposed Motorola G100 Android smartphone running proprietary software (version 5.0.0/4). The wearable displays can be set to show images from either the central head-mounted camera or the higher-resolution camera on the phone. Image input, magnification, brightness and contrast can be adjusted using the high contrast controls on the handset (Figs. 1, 2).

### Baseline Assessment

A baseline assessment gathered data about age, gender, ethnicity, UK sight loss registration status, number of years VI had affected life, and existing low vision solutions. All participants completed an initial low vision assessment, the format of which followed current UK best practice guidelines.^39^ This assessment was used to optimize spectacles, optical LVAs, participants' use of their existing technology, and to exclude secondary pathology. In addition, advice was offered about adaptations to lighting, color, and contrast.

Participants were introduced to the device to familiarize themselves with controls and performance in a standardized series of distance and near activities. Participants reaching basic competence levels were invited to take part in the home trial, and reasons for withdrawal at this stage were recorded.

### Intervention

A prospective, non-masked two-arm AB/BA crossover RCT was designed, with the protocol available online before recruitment.^40^ The crossover trial design was chosen because participants act as their own controls, reducing the sample size required to demonstrate an effect, and its use is recommended in studies where treatment is a minor modification of the control group.^41^ High dropout rate and carryover effect were not anticipated in this trial, because earlier studies have shown low levels of participant withdrawal,^16^^,^^24^ and wEVES were assumed to have no lasting effect when removed. A minor adverse event occurred early in recruitment, with a participant fainting. In response, exclusion criteria were altered from the published protocol to exclude those with balance-related disorders, heart disease, and uncontrolled high blood pressure. The cause was not directly attributable to the device, and no evidence of similar events was found in other trials or reported by local retailers. The length of the trial arms were shortened to allow sufficient data collection in the time available.

Intervention A was a six-week home trial with both wEVES and the participants’ existing low vision solutions, and intervention B was six weeks with existing low vision solutions only. Participants were randomized into the trial arms, with allocation determined using a web-based random plan generator (www.randomization.com). Allocation was on a 1:1 ratio with stratification by sex and binocular corrected distance visual acuity (≤1.00 logMAR, >1.00 logMAR). A repeating block size of 10 participants was chosen to ensure that available resources could meet the allocation generated. Because of their nature, wEVES were assumed to have no carryover effect when removed, and because the alternate intervention was “treatment as usual,” no washout period was deemed necessary.

**Figure 3.**
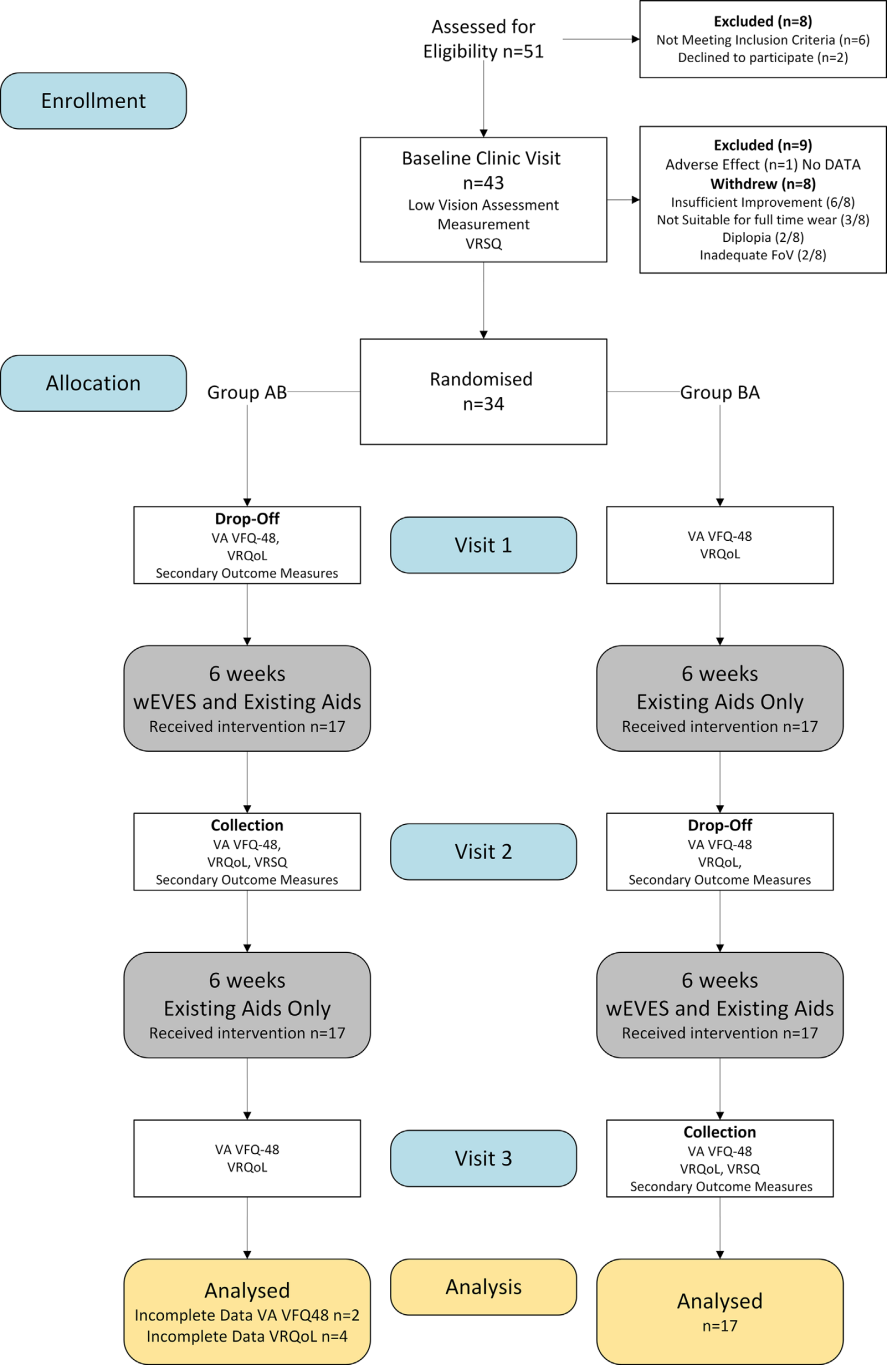
Flow chart allocation and dropout of participants from the two-armed crossover trial. CS, contrast sensitivity; FoV, field of view; VA, visual acuity.

Three home visits separated by approximately six weeks were undertaken with wEVES allocated at visit 1 and removed at visit 2 for the AB group. For the BA group, wEVES were dropped off at visit 2 and collected at visit 3 (see Fig. 3). Participants underwent a documented in-person three-stage training program to ensure their competency to safely and independently use the device. Participants were required to pass level 1 training at the baseline visit to be considered for inclusion in the home trial and level 2 before a device could be left with them for independent use at home. Level 3 training included additional items that were not essential for the day-to-day operation of the device (See Supplementary Information S1). Competency was deemed to be achieved when the participant was observed to complete the training activity without prompt from the researcher. Participants were supported by phone call advice or using additional face to face training sessions, which were provided as needed. The planned phone calls were used to answer questions and identify additional training needs during the intervention period, with scripted calls made in the control period to ensure both groups were given equivalent attention. Participants were encouraged to use the device throughout their daily lives but were asked not to use it for tasks involving movement. This restriction was due to the unknown risk of falling when using wEVES and manufacturers’ advice with similar devices.^42^^,^^43^ Measures of visual ability, preferred aid, and adverse effects were completed face-to-face. Measures of VRQoL were taken in prearranged phone calls before each home visit to reduce participant question load. The same researcher, an experienced optometrist (AM), collected all the data.

**Figure 4.**
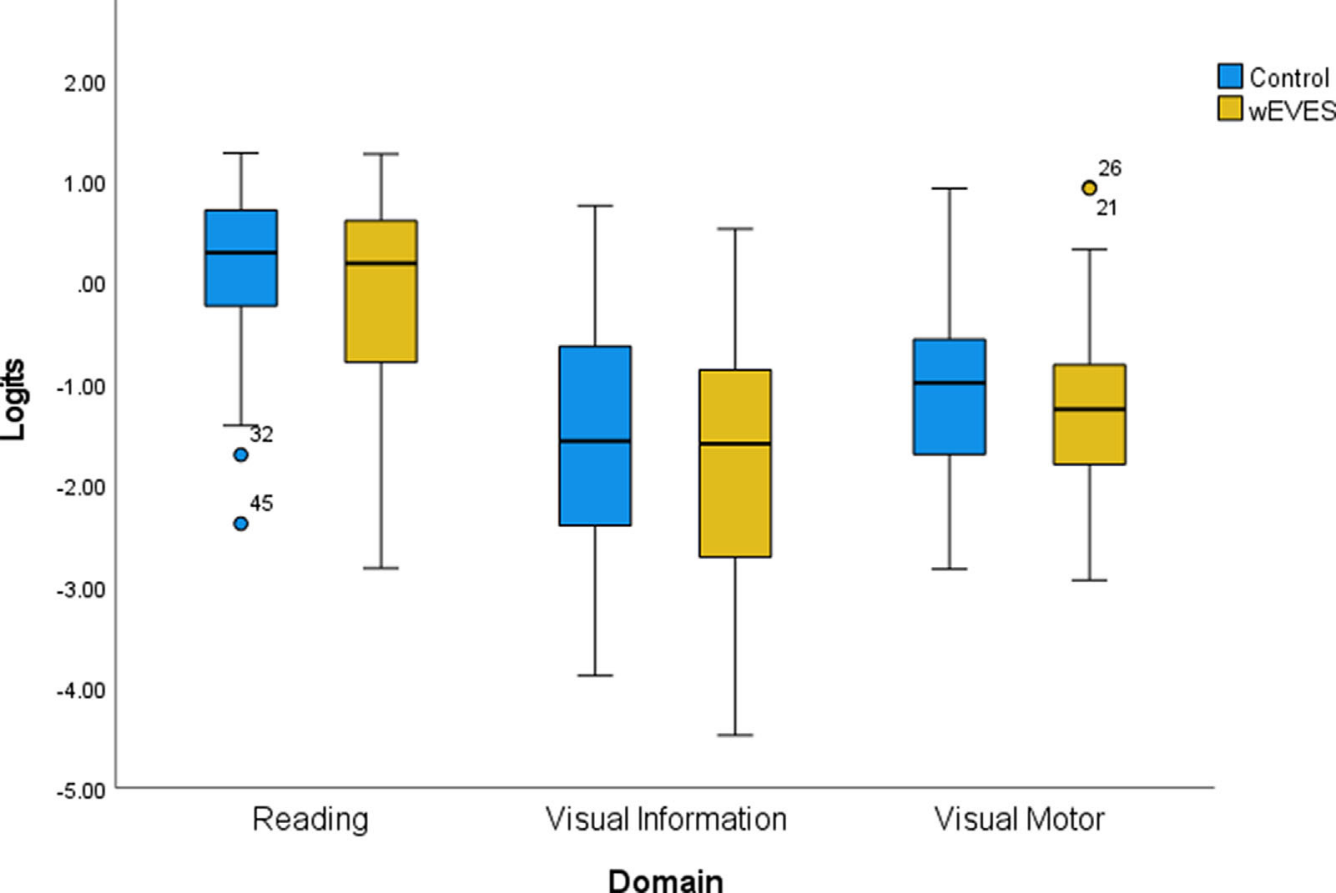
Graph showing the Logit values for wEVES and control, where the line represents the median value and the box IQR. Outlier numbers represent participant-allocated numbers.

Anglia Ruskin University granted ethical approval for the trial (ETH2223-3787), which conformed to the tenets of the Declaration of Helsinki. All participants provided written, informed consent, and the trial was registered with the ISRCTN registry (ISRCTN16865847) before enrolment commenced. Reporting the trial followed CONSORT 2010 statement: extension to randomized crossover trials^44^ (see Supplementary Information S2).

#### Primary Outcome Measures

The primary outcome measure was self-reported activity limitation, measured using the Veterans Affairs Low Vision Visual Functioning Questionnaire (VA-VFQ48)^45^^,^^46^ an instrument developed using Rasch analysis to give a measure of self-reported visual ability for adults with low vision. It has been used widely, including within studies assessing the benefits of wEVES^18^^,^^19^^,^^47^ and has demonstrated good psychometric properties.^48^ The test asks participants to rate the difficulty of completing 48 activities on a four-point Likert scale across four functional domains: reading, visual information, mobility and visual motor. Data was collected for all four domains, but the mobility data was not directly used in the analysis as the device was not used for these tasks. Before use, the 48 activities were assessed by the authors and an expert patient for their current suitability for use with a UK population. One activity, “Make out a check,” was considered outdated and changed to a similar short writing task, “Writing a birthday card.” The extended question set assessed self-reported task importance (three-point scale) and the participants’ preferred device to complete each task (see Table 2 and Supplementary Material S3).

**Table 2.** Hypotheses Under Investigation and Associated Primary Outcome Measures

Hypotheses Investigated	Primary Outcome Measure
Hypothesis 1. Reduce self-reported activity limitation	VA-VFQ48
Hypothesis 2. Be more versatile, allowing use for tasks without a current solution	VA-VFQ-48 extended questions
Hypothesis 3. Be the preferred LVA and be used more often than existing solutions	VA-VFQ48 extended questions and 5-level Likert Question
Hypothesis 4. Improve VRQoL	VisQoL and VCM-1
Hypothesis 5. Be a cost-effective solution in improving the VRQoL for people with AMD	VisQoL

To avoid duplication, two measurements of VRQoL were chosen that did not include elements of activity limitation. The VRQoL Core Measure (VCM-1) is a reliable unidimensional measure^49^^–^^51^ that can be administered over the phone.^52^ The Vision and Quality of Life Index (VisQoL) is a six-item questionnaire specifically designed to assess the impact of vision impairment on QoL for the economic evaluation of eye care and rehabilitation programs^53^ and has been used in studies assessing the cost-effectiveness of portable electronic vision enhancement systems.^54^ Any significant changes in VisQoL values due to wEVES could be used to generate quality-adjusted life year data, which can serve as a summary measure of health outcomes for economic evaluation.

#### Secondary Outcome Measures

The “Virtual Reality Sickness Questionnaire” (VRSQ)^55^ was used to assess the adverse effects of the wEVES. The VRSQ was specifically designed for Head-Mounted Displays and is a shortened version of the simulator sickness questionnaire^56^ used to assess adverse effects in flight simulators. Willingness to pay was explored using a question that enabled participants to understand the relative costs of other devices while removing real-world financial constraints that may influence their decision: “Your hand magnifier costs £50, an iPad costs £500, and a tabletop video magnifier costs approximately £2000. Imagining money was no problem; how much would you be willing to pay for this device?” Qualitative questions scored on a five-point Likert scale were tested at drop-off and collection of the wEVES to understand attitudes towards continuing to use the device, its simplicity, and ease of use. Data collection commenced on May 15, 2023, and ended on June 28, 2024.

### Data Analysis

The VA-VFQ48 and VCM1 patient-reported outcome measures (PROMs) were Rasch analyzed to convert ordinal responses to interval data.^57^ A single Andrich model was used in Winsteps (version 5.6.4.0; winsteps.com), with data stacked across time points. Person measures were derived in logits, representing the likelihood of a person having the ability to achieve an item or an item being achievable for a person.

The following parameters were examined to consider whether the Rasch model was acceptable, and all criteria were achieved unless specified in the results:

• Categories were ordered in terms of functional ability, with each category the most common response at some point on the functional scale.
• Individual items had fit values (infit and outfit) of <2 mnsq, indicating that no items had the potential to harm the scale.^58^
• Person separation value of >2.0 and reliability of >0.8 indicated an ability to discriminate between participants reliably.
• Item separation of >3.0 and reliability of >0.9 indicated that items were reliably ordered in terms of difficulty.^59^
• Adequate unidimensionality was indicated by more than 50% variance explained by the primary measure in principal components analysis and contrasts of less than 3 eigenunits.^60^

CROS t-test and regression analyses were performed to investigate the association between measurement changes across time points in keeping with the repeated measures crossover design.^61^^–^^65^ Data were analyzed using IBM SPSS statistics for Windows software (version 28.0; SPSS Inc., Chicago, IL, USA), and statistical significance was defined as *P* < 0.05.

## Results

Forty-two participants completed the baseline clinic assessment, 34 were randomized into the two arms of the trial, and eight participants withdrew from the trial (see Table 3). Multivariate analysis of variance test showed a significant difference between the participants who continued into the trial compared to those who withdrew (*V* = 0.34; *F*(4,37) = 4.67; *P* = 0.004). Older age (*F*(1,40) = 5.65; *P* = 0.02) and length of time (VI *F*(1,40) = 5.18; *P* = 0.03) were significantly associated with withdrawal but not distance VA with spectacles (*F*(1,40) = 2.40; *P* = 0.13) or wEVES (*F*(1,40) = 0.01; *P* = 0.92). Additionally, two-way χ^2^ tests showed no influence on withdrawal based on sex (χ^2^ = 0.14; *P* = 0.91), current use of smartphones (χ^2^ = 0.275; *P* = 0.60) or other non-wearable EVES (χ^2^ = 1.930; *P* = 0.17). Reasons for self-withdrawl were self-reported to include insufficient visual improvement noted to wish to continue (6/8), device not suitable for full-time wear (3/8), diplopia (2/8), and inadequate device field of view (2/8).

**Table 3.** The Demographic Data of the Study Participants in Arms AB, BA and Those Who Declined to Complete a Home Trial After the Initial Demonstration

	Group AB	Group BA	Withdrew at Initial Visit
n	17	17	8
Age, mean(SD)	80.9 (5.8)	79.6 (6.6)	**86.3 (7.5)** ^*^
Sex (% female)	70.5%	58.9%	62.5%
UK Registration Status			
SSI	4	4	2
SI	5	7	1
Not Registered	8	6	5
Months VI, Mean(SD)	76 (49)	61 (47)	**133 (135)** ^*^
BCVA logMAR Mean(SD)	0.80 (0.38)	0.83 (0.27)	0.59 (0.51)
General Health			
Very good	4	4	4
Good	5	6	2
Fair	8	7	2
Bad/Very Bad	—	—	—
Distance Optical LVA User	5/17	8/17	
Near Optical LVA User	17/17	17/17	
Smartphone User	82.40%	76.50%	87.50%
EVES/OCR User	52.90%	23.50%	12.50%
Residential status			
With sighted spouse	9	9	3
Alone	8	8	2
Supported living	—	—	2

BCVA, best corrected visual acuity following Subjective Refraction; EVES, Electronic Vision Enhancement device; OCR, optical character recognition; SI, Sight Impaired (<6/60 with full fields); SSI, severely sight impaired (<3/60 with full fields).

* Data significantly different to participants included in trial *P* < 0.05.

At baseline, all 42 participants reached the minimal level 1 of training competency to operate the device. The mean (SD) total training time to achieve level 2 competency for the 34 participants entering the home trial was 123 minutes (27), with two training sessions needed by 30/34 and a third face-to-face session required by 4/34 participants.

### Primary Outcomes

#### Activity Limitation

VA-VFQ48 item difficulty scores were Rasch analyzed for 32 participants, with 2 excluded because of incomplete datasets (See Table 4), to give a person score for visual ability, with more negative values indicating lower difficulty and therefore higher ability scores. Repeated-measures analysis of variance using a general linear model of VA-VFQ48 data showed that overall visual ability was significantly improved with wEVES than without (mean logit difference −0.26; 95% confidence interval [CI], −0.48 to −0.04; *F*(1,30) = 5.72; *P* = 0.02).

Group allocation did not affect outcomes (*F*(1,30) = 0.08; *P* = 0.79), indicating no carryover or period effect within the data. Analysis within the domains showed no significant differences in the reading (−0.39; 95% CI, 0.06 to −0.83; *t*(30) = −1.77; *P* = 0.09), visual information (−0.22; 95% CI, 0.13 to −0.58; *t*(30) = −1.27; *P* = 0.21), and visual motor (−0.22; 95% CI, 0.09 to −0.52; *t*(30) = −1.46; *P* = 0.16) domains (see Fig. 4).

**Table 4.** Raw Data From the Two Arms of the Trial, AB and BA, Taken at Visit 2 and Visit 3

			Intervention			Control		
				95% CI for Mean			95% CI for Mean	
PROM	Arm	n		Lower	Upper		Lower	Upper
VA-VFQ48			Mean logits			Mean logits		
	AB	15	−0.858	−1.303	−0.413	−0.739	−1.161	−0.318
	BA	17	−0.944	−1.374	−0.514	−0.802	−1.168	−0.437
VCM-1			Mean logits			Mean logits		
	AB	13	1.330	0.700	1.960	1.532	0.703	2.362
	BA	17	1.828	1.216	2.439	1.530	0.949	2.111
VisQoL			Mean utility weight			Mean utility weight		
	AB	13	−2.158	−3.183	−1.134	−1.843	−2.735	−0.951
	BA	17	−1.925	−2.713	−1.138	−1.930	−2.809	−1.051

CI, confidence intervals; PROM, patient reported outcome measure; VA-VFQ48, veterans affairs low vision visual functioning questionnaire; VCM-1, VRQoL core measure; VisQoL, vision and quality of life index.

VA-VFQ48 data were racked, and Rasch analyzed to identify any individual items changing significantly above normal background variation. Changes in item logit measure were compared to those noted in items where wEVES were not used (mean change −0.09logits [SD = 0.47]). The only item showing >1.96 SD of improved visual ability greater than the background was “Recognizing faces across the room” (0.85 logits).

Based on findings from previous research,^31^ a linear model using a two-block hierarchical regression was used to understand whether age, smartphone usage, VA with wEVES, or sex, baseline contrast sensitivity and VRSQ predicted the changes in visual ability. Neither block 1 (*R*^2^ = 0.039; *F*(3,28) = 0.38; *P* = 0.77) nor block 2 (*R*^2^ = 0.066; *F*(3,25) = 0.38; *P* = 0.87) showed a significant explanation of the linear model, with results summarized in Table 5.

**Table 5.** Linear Model of Predictors of the Change in VA-VFQ48 Score

Block	b	SE B	β	*P* Value
1				
Constant	−0.002	0.865		0.996
95% CI	−1.301 to 1.200			
Age	−0.002	0.010	−0.042	0.772
Smartphone User	−0.086	0.148	−0.119	0.482
Distance VA wEVES	0.247	0.340	0.140	0.521
2				
Constant	0.266	0.985		0.789
95% CI	−1.581 to 2.078			
Age	−0.001	0.011	−0.019	0.924
Smartphone User	−0.091	0.171	−0.127	0.562
Distance VA wEVES	0.073	0.512	0.042	0.907
Sex	−0.048	0.136	−0.079	0.755
CS	−0.103	0.264	−0.109	0.777
VRSQ	0.024	0.034	0.141	0.468

CS, contrast sensitivity; VA, visual acuity.

CI and SE based on 1000 Bootstrap Samples.

#### WEVES Versatility

**Figure 5.**
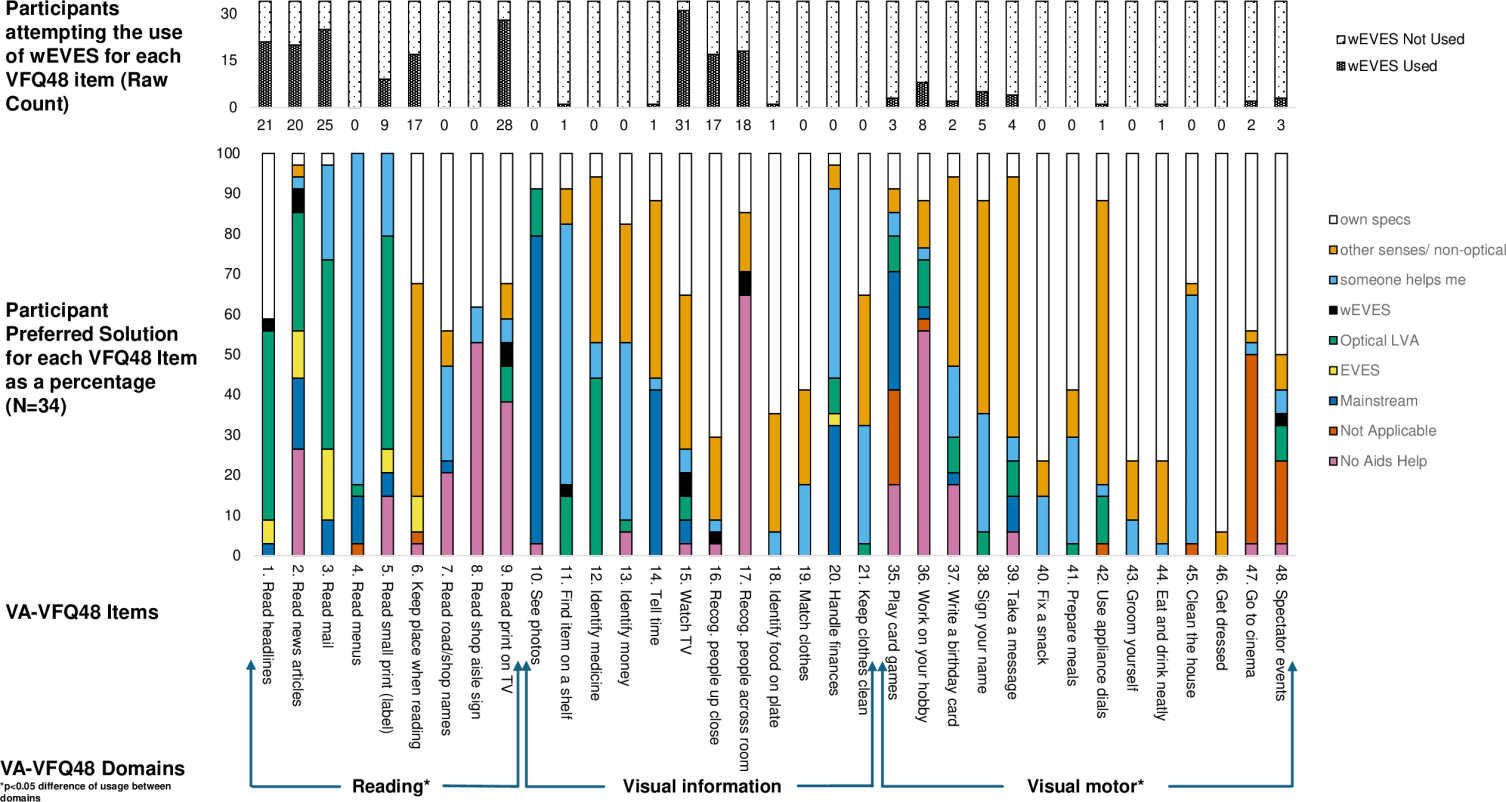
Participants' attempted use of wEVES and responses at the end of the wEVES trial to which aid would be preferred to help with each item from the VA-VFQ48. Specs, spectacles; EVES, (non-wearable) electronic vision enhancement systems; mainstream, Smartphone or tablet used as an LVA; Recog, recognize.

Participants reported attempted use of the wEVES for 21 out of the 35 VA-VFQ48 items, with the mean(SD) number of items attempted per participant being 6.4(2.6). One-way analysis of variance of the VA-VFQ48 showed significant differences in attempted usage of wEVES between the three domains (*F* 2,32 = 4.92 *P* = 0.01). The greatest variety of attempted wEVES use was in the reading (39.2%, 95% CI [64.7, 13.7]) and visual information domains (16.9%, 95% CI [36.2, −2.4]), with significantly lower levels of use in the visual motor domain (6.1%, 95% CI [10.1, 2.07]). The individual items with the greatest percentage of participants attempting usage were watching TV (91.1%), reading mail (73.5%), recognizing people across the room (53%) and recognizing people up close (50%). Lesser attempted use was noted with hobby work (23.5%) and signing your name (14.7%) (see Fig. 5).

#### Preferred Solution

After the wEVES arm of the trial, participants were asked what their preferred low vision solution was for each of the VA-VFQ48 items, and data was analyzed for all 34 participants. The wEVES was not the preferred device to use for any visual task across people, or across tasks for any person. The wEVES was the preferred solution for reading newspaper headlines, reading print on TV, watching TV and recognizing people across the room for 2/34 participants. Additionally, the wEVES was the preferred solution for 1/34 people reading newspaper headlines, finding something on a shelf, recognizing people up close and going to spectator events (see Fig. 5).

**Figure 6.**
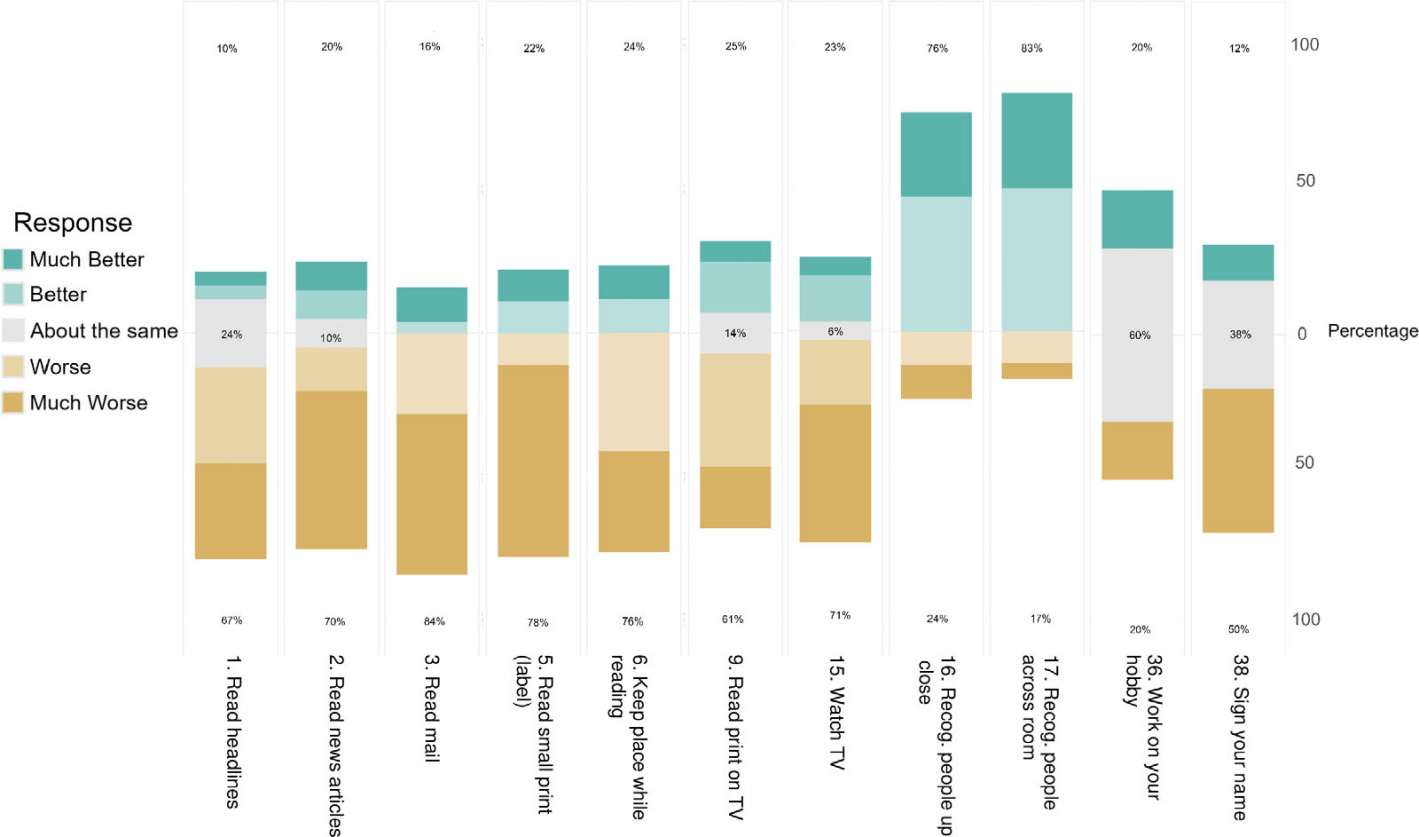
Participants' responses showing how wEVES compared with their best alternative solution for each task. Only items with greater than five responses are shown. Recog, recognize. Percentages represent the proportion of detracting, neutral, and positive statements for each question. (Graphing JASP 19.1.0).

For each of the VA-VFQ48 items, participants were asked how wEVES compared to their best alternative solution. The wEVES was rated as worse or much worse than existing low vision solutions in all categories by the majority of users, except for the items relating to recognizing faces. For the items, recognizing people up close (13/17) and recognizing people across the room (15/18), wEVES were rated as better or much better than their alternatives by the majority of participants (see Fig. 6).

#### VRQoL

Data for 30 participants were analyzed, with four participants in the AB group declining to complete the QoL questionnaires at visit 3 because of discomfort in answering further questions relating to quality of life (see Table 4). It was speculated that the higher rate of incomplete data in group AB was due to participants considering that the intervention element of the trial had been completed when the wEVES was removed at visit 2, making them more reluctant to answer additional questions at visit 3.

Before analysis, ordinal data for the VisQoL were transformed into estimates of utility weights using the standard algorithm,^66^ whereas VCM-1 ordinal data were Rasch analyzed to provide scale person measures. Data from both questionnaires showed no carryover effect, but neither VisQoL (mean difference = 0.02; 95% CI, −0.01 to 0.04; *F*(1,28) =1.20; *P* = 0.28) nor VCM-1 (mean difference = 0.10; 95% CI, −0.27 to 0.46; *F*(1,28) =0.292; *P* = 0.59) data showed a significant change because of the intervention. Because of the nonsignificant findings, no analysis of the cost-effectiveness of VRQoL was completed.

### Secondary Outcomes

#### Willingness to Pay

**Figure 7.**
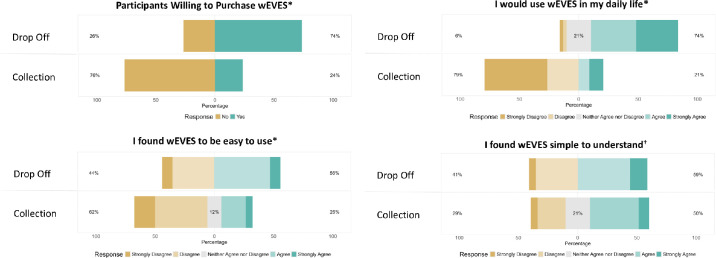
Participants' responses to statements at the drop-off and collection of wEVES. Willingness to purchase wEVES (yes/no); If available, I would use wEVES in my daily life (5-level Likert); I found wEVES to be easy to use (5-level Likert); I found wEVES simple to understand (5-level Likert). Where percentages represent the proportion of detracting, neutral and positive statements for each question. *P < 0.001, †*P* < 0.05, representing a significant difference between drop-off and collection of the device. Graphing JASP 19.1.0.

The number of people willing to purchase the device fell from 25/34 at drop-off to 8/34 at collection of the device. Wilcoxon signed-rank test showed the amount that people were willing to pay was significantly lower at collection (median = £0; interquartile range [IQR] 5; mean [SD] = £105 [359]) than at drop-off (median = £300; IQR 1500; mean [SD] = £759 [1018]; *T* = 10, *P* < 0.001, effect size *r*= −0.49). Similarly, users’ perceptions of the device's simplicity (*T* = 61.5, *P* = 0.01, *r*= −0.30), ease of use (*T* = 14, *P* < 0.001; *r* = −0.50) and imagined daily usage of the device (*T* = 0; *P* < 0.001; *r* = −0.60) all showed significant decline between drop-off and collection of the device (see Fig. 7).

#### Adverse Effects

**Figure 8.**
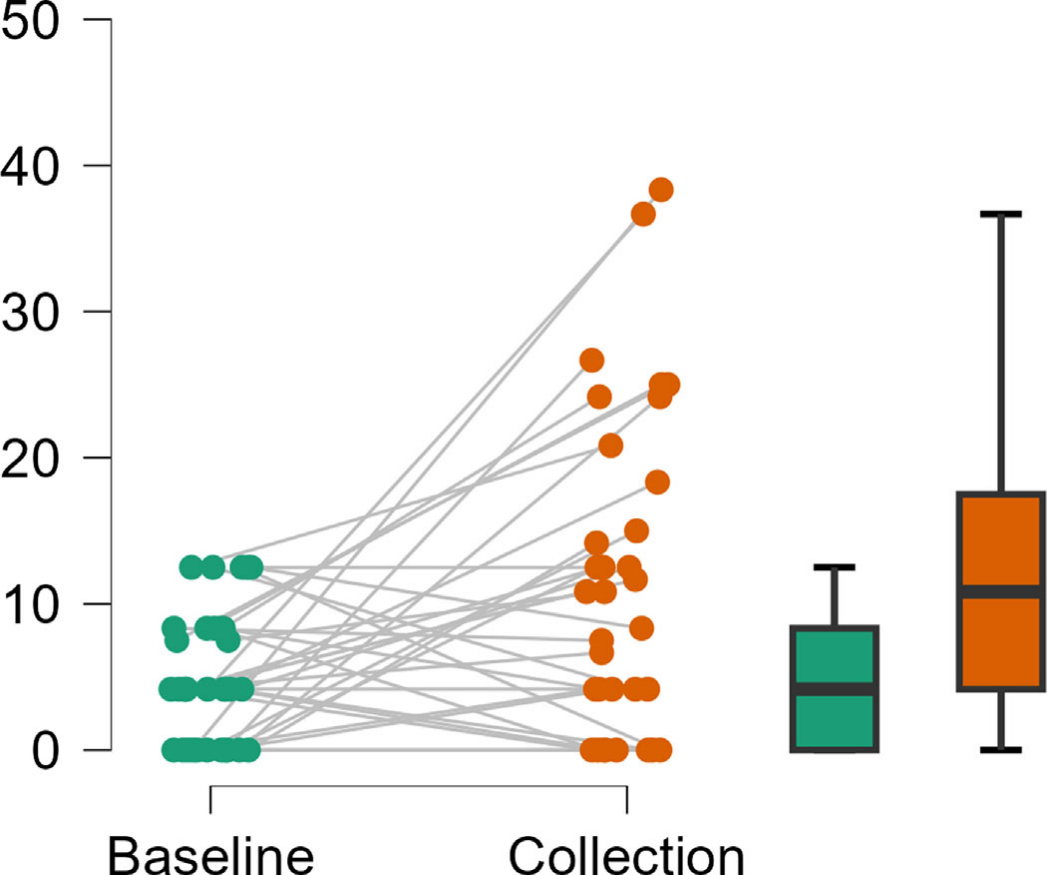
Raincloud plot of transformed VRSQ Scores^67^ shown at the baseline clinic visit and collection of the device. Where the color *green* represents data at the initial baseline visit and *orange* represents data at collection. *Dots* represent individual data points with lines indicating paired data. Box plots show median, IQR, and range of data. Graphing JASP 19.1.0.

Rasch analysis of VRSQ data showed low person reliability, poor unidimensionality and targeting with many scores of the minimum value. Therefore VRSQ data was analyzed non-parametrically after data conversion using the recommended standard algorithm and scoring strategy.^55^^,^^67^ The converted VRSQ is scored in a range from 0 to 100, median recorded scores indicated low levels of reported symptoms at both the initial trial of the device in the clinic (median = 4.2; IQR 8.3) and collection (median = 10.8; IQR 13.3). Wilcoxon signed rank test showed VRSQ results were significantly higher at collection than at initial clinic trial, *T* = 351; *P* = 0.004; effect size *r* = 0.35), but nonparametric analysis using Kendall's tau showed no correlation between the total measures taken at the two time points (*T* = −0.03; BCa CI, −0.31 to, 0.23; *P* = 0.83) (see Fig. 8). Further analysis showed the change in noted symptoms was driven by significant changes in the disorientation domain (*T* = 120; *P* < 0.001; effect size *r* *=* 0.42) as opposed to the oculomotor domain (*T* = 258; *P* < 0.09) of the VRSQ. When noted, the majority of symptoms were “slight,” with the most common items being general device discomfort (21/34), followed by fatigue (11/34) and headache (10/34). However, adverse effects were enough to cause two users to discontinue usage, and 9/42 participants reported some instances of diplopia when using the device for near tasks, which may have been due to an inability to adjust screen separation on the tested device. No lasting adverse effects were reported and none of the devices required replacement or exchange because of breakages or malfunction.

## Discussion

This investigation presents results from a home trial exploring the usefulness of a non-enclosed wEVES for people with AMD. Earlier studies have tended to use prospective observational intervention designs in mixed populations. However, the best practice recommendation for the trial of virtual reality devices in medicine is to use RCT with a comparison between intervention and control.^32^ Our randomized crossover study design with a homogenous ocular pathology has allowed participants to act as their own controls and enhance the quality of the data found.

Results showed that the device significantly improved the overall measure of visual ability but not two measures of VRQoL. Participants attempted using wEVES across a range of distance and near tasks, but they were not the most preferred choice of aid for any individual or activity. Additionally, user satisfaction and willingness to use or purchase the device fell significantly during the trial.

### WEVES Effect on Self-Reported Activity Limitation

Results in this population of users with AMD echoed others' findings in mixed populations, demonstrating that using wEVES led to improvements in measures of self-reported visual ability.^18^^,^^24^^,^^47^^,^^68^ However, in previous studies, the improvement in vision-related activity was primarily driven by changes in the reading domains of the PROMs,^18^^,^^24^^,^^68^ in contrast, data here suggest that improvements in facial recognition were the most significant factor. Qualitative data from the same sample revealed dissatisfaction with the performance of the device tested, particularly with the limited field of view and unsatisfactory image quality for reading tasks.^69^ Further work investigating the perceptions of a younger sample towards the same device would be needed to understand if the difference is caused by the device tested or the attitudes of an older population adapting to new technology.

Many studies report that wEVES significantly improved baseline measures of both distance and near acuity,^15^^–^^20^^,^^70^ a fact echoed by a subset of the first 30 users investigated here.^71^ Visual function and visual ability have routinely been used as the primary outcome measures in research to assess the utility of wEVES.^16^^–^^18^^,^^20^^,^^22^^,^^24^^,^^68^ Given the findings of reproducible improvements in measures of objective visual function and subjective activity in this data, it may be reasonable to conclude that wEVES are effective and, by extension, useful to those with AMD. However, to infer usefulness solely from numeric evidence of improvement in activity and acuity would be to reduce the effects of VI to a simplistic medical model where the sight loss is seen as a set of measurable attributes needing to be increased to remedy the broken sense. Viewing sight loss within this restricted paradigm disregards the importance of the social model of sight loss, which advocates that disability arising from VI results from societal barriers limiting inclusion and involvement rather than the sensory loss itself.^72^^,^^73^ Abandonment of wEVES is not solely driven by performance but has other contributory factors, including the device's weight,^17^^,^^21^^,^^74^ handling,^18^ the view of others,^15^ adverse effects,^17^^,^^21^^,^^24^ and the confined nature of the enclosed devices.^75^ For this reason, it is recommended that in addition to conventional measures of activity and functional improvement, a broader range of qualitative factors are considered, which, when viewed together, will better indicate the sustained usefulness of wEVES for people with AMD.

### WEVES Versatility and Use for Tasks Without a Current Solution

Our findings showed attempted use of wEVES across all three domains of the assessed VFQ48 but with the greatest attempted use for reading tasks. This finding highlights not only the potential versatility of a single device to provide multiple solutions but also emphasizes the importance of reading activities for participants with AMD. The significance of reading is noted in observational studies^76^ and surveys^77^ involving wEVES alongside investigations reporting the broader rehabilitation needs of individuals with VI.^78^^–^^80^ However, reading is an area where people with AMD already use a wide range of existing coping strategies with proven effectiveness.^8^^,^^81^^,^^82^

Our data showed that many of the individual items of greatest interest for users to try wEVES were distance tasks such as television and facial recognition. In this sample, “Watching the TV” was the item with the largest attempted use, showing consensus with the importance of this task reported within data from mixed populations.^17^^,^^24^^,^^76^^,^^83^ Confoundingly, facial recognition has been reported as a main priority in a survey of 50 people with VI (n = 11 AMD)^77^ but ranked only forty-second out of 56 tasks for importance by observation of 32 visually impaired users (n = 7 AMD).^76^ The divergent needs and priorities of people with different ocular pathology and demographics may account for the disparity. Our findings in a more homogeneous population of older adults with similar pathology show the importance of distance as well as near solutions for this group. Prescribing of distance telescopic devices is low compared to near LVA and the abandonment of these devices is higher than other aids.^84^^,^^85^ There are significant challenges in prescribing suitable solutions that are able to deliver contrast enhancement and magnification for visual information tasks, which is highlighted in our results showing that many participants currently used no assistive aid to complete distance tasks. Therefore, if effective and suitable, wEVES could bridge this gap to support these unmet needs of people with AMD.

### Preferred Low Vision Solution

After the initial demonstration, 81% (34/42) of participants were willing to undertake a home trial, indicating the desirability of wEVES as a concept for people with AMD. This figure is notably larger than the 47% (28/60) of a mixed population willing to use an enclosed style wEVES following a similar demonstration with comparable visual improvements.^17^ Convenience sampling in the current study may account for some of the asymmetry, but this difference may reinforce the suggestion that enclosed wEVES are less aesthetically desirable than spectacle-style non-enclosed wEVES.^75^

Self-reported visual ability data measured whether a device improved a participant's capacity to complete a task. In contrast, the preferred solution data reflected whether the participant was willing to use the device to achieve the measured benefit. Our data showed very low acceptance of a wEVES as a preferred device for any task. Tasks such as recognizing faces showed improvement in self-reported ability as well as subjective approval as performing better than the available alternatives. However, the underlying compromises that participants identified with the use of a wEVES meant they consistently chose not to use the device for this task. These findings indicate there are broader drivers to success than addressing the functional gaps, with previous studies suggesting that successful wEVES use is related to higher satisfaction with technology and improvements in functional independence.^21^ Qualitative results from this trial indicated that the compromises driving the participants’ reluctance to use the device resulted from the performance of the tested device, as well as practicality barriers within the current technology, which may be more attributable to the wider genre of wEVES.^69^ These compromises mean that, despite improvements in visual ability, the device tested was not perceived as a step-up compared to the status quo*.*

The competitive enablement model used in the study required participants to explore wEVES in user-defined tasks rather than restricting their decisions to controlled, investigator-led activities. When evaluating different optimal solutions, a wearable device has obvious benefits compared with other low vision aids, as it provides a portable and hands-free solution. Conversely, wearable devices also have recognized inherent challenges compared with other solutions in attempting to deliver low-latency, stabilized images of adequate resolution, contrast and field.^17^^,^^24^^,^^33^^,^^86^ The CE concept additionally necessitates that participants compare wEVES with their existing coping strategies, and the preferred solution data differs from and extends the work of others who have used home trials to evaluate wEVES’ impact on visual ability compared to a baseline.^15^^,^^16^^,^^18^^,^^24^^,^^70^ Some may view this approach as introducing bias by assessing participants across non-standardized activities with user preference toward established solutions over new ones. However, users already use a mix of prescribed and improvised aids, and wEVES must prove their value within this competitive landscape to become the preferred option. Discontinuance in use of AT is common and can be driven by the need for replacement or disenchantment with current technology.^21^^,^^87^^–^^89^ Within this data, user-identified compromises in performance and practicality mean that most participants tended not to use a wEVES, even for tasks where they showed improvements in both subjective and objective measures of functional vision.

### WEVES Effect on VRQoL

Findings showed no significant improvement in two VRQoL measures, contrasting with a Canadian study where 57 participants using the non-enclosed eSight device reported an increase in VRQoL.^47^ A separate study of 109 eSight users linked higher VRQoL with sustained device use,^21^ which may explain the lack of improvement in our sample with little interest in ongoing use. A South Korean trial with an enclosed device showed VRQoL improvement only in a subset of participants under 40 years old,^16^ and a Chinese study of 41 users of a non-enclosed wEVES found a negative correlation between VRQoL improvement and age.^90^ These findings suggest a greater impact of the current technology amongst younger users and indicate the need to engage with older adults separately to better understand the facilitators and barriers to their sustainable use of a device, which they initially found appealing.

### Additional Outcomes

The training data revealed that all participants achieved basic competency with the device after two or three sessions, suggesting the suitability of this type of device for use with an older demographic with varied levels of proficiency with technology. However, it also underlines the necessity for ongoing practitioner involvement with users after issuing devices. Telerehabilitation has shown promise in providing training with wEVES for a younger cohort with mixed pathology^47^^,^^91^ and smartphone app training for an older cohort of adults with VI.^27^ However, further research is required to determine whether telerehabilitation would be suitable for the specific needs of older adults with AMD using wEVES for the first time.

The number of participants willing to purchase the device was initially high but fell significantly by the end of the home trial. Direct measurement of willingness to purchase can be problematic,^92^ but the findings are in keeping with other studies that demonstrated the amounts willing to be paid decreased during a trial^22^ and were at all times significantly below the device's retail cost (GBP 3999).^22^^,^^24^ Initial qualitative questions indicated a device participants felt was simple to understand and easy to use, and it may be anticipated to see an increase in user confidence with increased familiarity with the technology. In contrast, our data showed self-reported ease of use and perceived simplicity decreased as participants' perceptions shifted during real-world tasks compared to the initial controlled demonstrations. When evaluating wEVES, ease of use has been noted to be as important to users as visual improvement,^93^^,^^94^ further explaining the decrease in willingness to purchase. The evidence of changing attitudes of participants towards a wEVES over time would caution individuals seeking to prescribe or purchase a device who see an improvement in visual function and functional ability following a brief clinical demonstration. Longer home trials in user-selected activities are recommended to more accurately assess the sustained usefulness of a new device.

In common with adverse effects measured with both enclosed^16^^,^^17^^,^^24^^,^^68^ and non-enclosed wEVES,^47^ data suggested symptoms tended to be of a low level. Contrary to earlier studies,^24^^,^^47^ findings suggest a significant increase in symptoms between the start and end of the trial, with no correlation of symptoms noted between the two time points. Therefore an absence of symptoms during the short trial did not indicate the presence or levels of adverse effects following prolonged use. Some laboratory-based tests showed that people with AMD may experience simulator sickness differently from those with full sight.^95^^,^^96^ Therefore more work is needed to determine whether the difference from earlier findings is peculiar to the device under test or the older population under investigation.

## Reflections and Limitations

The described trial does not meet the gold standard of a double-masked RCT. Because of the nature of the device under investigation, it was impossible to mask the intervention group to the presence of the device, creating a potential bias between the arms of the study. Limited resources meant a single investigator completed all allocation and data collection. Although this created consistency in the application of the self-reported questionnaires, it was not possible to mask the data collection to the arm of the trial, creating potential for bias that could have been eliminated by using a second masked investigator.

Our convenience sample would suggest the inclusion of participants who may be more open and receptive to the idea of wEVES than the population they represent. Additionally, the trial protocol ensured that the participants all received updated LVAs, aligning with a UK National Health Service low vision assessment. Although this ensured a cohort with a range of competing solutions, it may have produced findings at odds with those from a sample who were device naïve without developed alternatives. However, this trial was conceived to provide pragmatic evidence to aid clinicians' prescribing decisions. Specifically, the trial was designed to mimic the real-world scenario in which new AT is introduced to a receptive population seeking to evaluate its benefits relative to their existing low vision solutions.

The primary outcome measures were assessed using PROMs whereas others have used cameras^76^ or data trackers^97^^,^^98^ to gather objective data on device usage. Access to app information to objectively assess the number, length, and times of use was prohibited by the standard device purchase agreement but this data could have added to the findings. A key strength of this study is its independence from manufacturers, reducing the potential for bias when evaluating the device in situ. However, because the assessment relied on subjective data from patient experiences, cooperative work with the manufacturers would potentially have allowed access to data that may have further enhanced the measured outcomes.

Where possible we have tried to draw parallels between our findings and the work of others who have used a variety of devices in different locations. However, further work is needed to assess what proportion of our findings are driven by the idiosyncrasies of the tested device and what is transferable to other devices of either the same or different styles. Additionally, further research is needed to determine whether these views hold across different countries and cultural contexts.

## Conclusions

Participants showed significant improvement in self-reported visual ability using a wEVES, indicating the potential for these devices to support visual rehabilitation. Additionally, users were willing to trial a non-enclosed wEVES for a wide range of tasks, including distance tasks, for which they reported having few alternative solutions. However, these noted improvements did not lead to changes in measures of VRQoL for people with AMD. Crucially, the wEVES did not consistently displace established solutions as the preferred device for completing tasks, even when their performance was self-reported as superior. Consequently, professionals should not rely solely on subjective and objective measures of visual function and ability to determine the likely benefits of assistive technology, as other broader factors drive sustained usefulness.

Finally, participants’ initially positive opinions toward this wEVES declined significantly after a home trial using the device for a wide range of user-selected activities. Therefore user-led trials are more indicative of the usefulness of wEVES for people with AMD than a short demonstration within a low vision clinic or retail display.

## Supplementary Material

Supplement 1
