# Supplementary material for: The Usefulness of a Wearable Electronic Vision Enhancement System for People With Age-Related Macular Degeneration: A Randomized Crossover Trial
Source: Transl Vis Sci Technol. 2025 Sep 4;14(9):8. doi: 10.1167/tvst.14.9.8 (PMC12416520; doi:10.1167/tvst.14.9.8)
Supplement: Supplement 1 [file tvst-14-9-8_s001.pdf]

Supplementary Information S1

Training competency framework

		Visit 1 Pass/Fail	Visit 2 Pass/Fail	Visit 3 Pass/Fail
**Time TAKEN**				
**Basic:** Pass this to complete the supervised trial in the clinic	Enter and Exit Sleep Mode			
	Put device on and take off			
	Use manual zoom-tactile buttons			
	Use manual zoom-screen control			
	Direct phone and head to items of interest			
	Plug in and Charge the Device			
	Understand When NOT to use the device*			
**Intermediate:** Participants must pass this to allow the home trial to commence	Connect/disconnect phone and specs			
	Turn on Brightext			
	Adapt Image Contrast			
	Adjust Screen brightness			
	Enable Eye switch. Phone and specs			
	Understand Basic Troubleshooting			
	Understand Clean and storage			
	Understand not to remove protective film			
	Repeat when NOT suitable to use a device*			
	Turn on Power save mode			
	Turn the device fully on and off			
	Not to add own SIM to phone			
**Advanced:** Additional settings for the more capable user but not essential for trial design	Set up Favourites settings			
	Set and Enable Autozoom			
	Enable Reference Line			
	Change Stabilisation settings			
	Use Portal Magnification			

*Walking in device, operating machinery, controlling moving vehicles, observing the sun or other solar events.

Supplementary Information S2

CONSORT 2010 statement: extension to randomised crossover trials

Section/topic	Item No	Description	Page No*
Title†	1a	Identification as a randomised crossover trial in the title	1
Abstract†	1b	Specify a crossover design and report all information outlined in table 2	2-3
Introduction:			
Background‡	2a	Scientific background and explanation of rationale	4-6
Objectives‡	2b	Specific objectives or hypotheses	Table 2 Page 12
Methods:			
Trial design†	3a	Rationale for a crossover design. Description of the design features including allocation ratio, especially the number and duration of periods, duration of washout period, and consideration of carry over effect	8
Change from protocol‡	3b	Important changes to methods after trial commencement (such as eligibility criteria), with reasons	8
Participants‡	4a	Eligibility criteria for participants	Table 1 Page 6
Settings and location‡	4b	Settings and locations where the data were collected	Fig 3
Interventions†	5	The interventions with sufficient details to allow replication, including how and when they were actually administered	Page 8-13 Fig 3
Outcomes‡	6a	Completely defined prespecified primary and secondary outcome measures, including how and when they were assessed	Fig 3, Table 2 and Online Study Protocol[40]
Changes to outcomes‡	6b	Any changes to trial outcomes after the trial commenced, with reasons	None
Sample size†	7a	How sample size was determined, accounting for within participant variability	Online Study Protocol[40]
Interim analyses and stopping guidelines‡	7b	When applicable, explanation of any interim analyses and stopping guidelines	None
Randomisation:			
Sequence generation‡	8a	Method used to generate the random allocation sequence	8
Sequence generation‡	8b	Type of randomisation; details of any restriction (such as blocking and block size)	8
Allocation concealment mechanism‡	9	Mechanism used to implement the random allocation sequence§ (such as sequentially numbered containers), describing any steps taken to conceal the sequence until interventions were assigned	27
Implementation†	10	Who generated the random allocation sequence,§ who enrolled participants, and who assigned participants to the sequence of interventions	8
Blinding‡	11a	If done, who was blinded after assignment to interventions (for example, participants, care providers, those assessing outcomes) and how	27
Similarity of interventions‡	11b	If relevant, description of the similarity of interventions	n/a
Statistical methods†	12a	Statistical methods used to compare groups for primary and secondary outcomes which are appropriate for crossover design (that is, based on within participant comparison)	12-13
Additional analyses‡	12b	Methods for additional analyses, such as subgroup analyses and adjusted analyses	12-13
Results			

Section/topic	Item No	Description	Page No*
Participant flow (a diagram is strongly recommended)†	13a	The numbers of participants who were randomly assigned, received intended treatment, and were analysed for the primary outcome, separately for each sequence and period	Fig 3
Losses and exclusions†	13b	No of participants excluded at each stage, with reasons, separately for each sequence and period	Fig 3
Recruitment‡	14a	Dates defining the periods of recruitment and follow-up	12
Trial end‡	14b	Why the trial ended or was stopped	12
Baseline data†	15	A table showing baseline demographic and clinical characteristics by sequence and period	Table 3
Numbers analysed†	16	Number of participants (denominator) included in each analysis and whether the analysis was by original assigned groups	Fig 3
Outcomes and estimation†	17a	For each primary and secondary outcome, results including estimated effect size and its precision (such as 95% confidence interval) should be based on within participant comparisons. In addition, results for each intervention in each period are recommended	Table 4 and results 13-20
Binary outcomes‡	17b	For binary outcomes, presentation of both absolute and relative effect sizes is recommended	n/a
Ancillary analyses‡	18	Results of any other analyses performed, including subgroup analyses and adjusted analyses, distinguishing prespecified from exploratory	13-20
Harms†	19	Describe all important harms or untended effects in a way that accounts for the design (for specific guidance, see CONSORT for harms32)	7<br>19-20
Discussion:			
Limitations†	20	Trial limitations, addressing sources of potential bias, imprecision, and if relevant, multiplicity of analyses. Consider potential carry over effects	26-27
Generalisability‡	21	Generalisability (external validity, applicability) of the trial findings	26-27
Interpretation‡	22	Interpretation consistent with results, balancing benefits and harms, and considering other relevant evidence	20-26
Other information:			
Registration‡	23	Registration number and name of trial registry	8
Protocol‡	24	Where the full trial protocol can be accessed, if available	8
Funding‡	25	Sources of funding and other support (such as supply of drugs), role of funders	1<br>28

Home Visit 1: Existing Aids

Manufacturer	Name	Power

A	1	Patient ID:				Date of Visit:					2	0		

Reading

Column A "I want you to tell me how difficult it may be for you to do the following activities. The task may be:
1=NOT difficult, 2=MODERATELY difficult, 3=EXTREMELY difficult, or 4= IMPOSSIBLE.
9= Not be able to do the task for a reason not related to your vision loss.

Column B "I want you to think about how important it is for you is to be able to do this task without help from another person. You can answer:
1= Not important, 2=Moderately important 3=Very Important"

Column C "I want you to think about what items you might use to help you do this task."
1=own specs 2=other senses/ non-optical 3=someone helps me 4=wEVES 5=Optical LVA 6=EVES 7=Mainstream 9=cannot complete with any aids

Column D "If someone else helps with the task, estimate how much help."
1=A lot, 2=50/50, 3=A little

Column E At Final wEVES VISIT ONLY ASK "Compared to next best solution wEVES are…"
1=Much worse, 2=Worse, 3=About the same, 4=Better 5=Much better
You can also say 0=Cannot Use wEVES for this task or 9="Only thing that I can use."

"These questions relate to reading and near vision activities. Remember, if you use a low vision device or adaptive technique to assist with the activity, please respond as though you were using the device or technique".

	A	B	C	D	E
1. Read newspaper headlines					
2. Read newspaper or magazine articles					
3. Read mail					
4. Read menus					
5. Read small print on a package label					
6. Keep your place while reading					
7. Read road signs and shop names					
8. Read signs (e.g., grocery store aisle)					
9. Read print on TV					

A	2	Patient ID:				Date of Visit:					2	0		

Visual information

Column A "I want you to tell me how difficult it may be for you to do the following activities. The task may be:
1=NOT difficult, 2=MODERATELY difficult, 3=EXTREMELY difficult, or 4= IMPOSSIBLE.
9= Not be able to do the task for a reason not related to your vision loss.

Column B "I want you to think about how important it is for you is to be able to do this task without help from another person. You can answer:
1= Not important, 2=Moderately important 3=Very Important"

Column C "I want you to think about what items you might use to help you do this task.
1=own specs 2=other senses/ non-optical 3=someone helps me 4=wEVES 5=Optical LVA 6=EVES 7=Mainstream 9=cannot complete with any aids

Column D If someone else helps with the task, estimate how much help.
1=A lot, 2=50/50, 3=A little

Column E At Final wEVES VISIT ONLY ASK "Compared to next best solution wEVES are…"
1=Much worse, 2=Worse, 3=About the same, 4=Better 5=Much better
You can also say 0=Cannot Use wEVES for this task or 9="Only thing that I can use."

"These questions relate to Visual information activities. Remember, if you use a low vision device or adaptive technique to assist with the activity, please respond as though you were using the device or technique".

	A	B	C	D	E
10. See photos					
11. Find something on a crowded shelf					
12. Identify medicine					
13. Identify money					
14. Tell time					
15. Watch TV					
16. Recognise people up close					
17. Recognise people from across the room					
18. Identify food on a plate					
19. Match clothes					
20. Handle finances					
21. Keep clean/keep your clothes clean					

A	3	Patient ID:				Date of Visit:					2	0		

Mobility

Column A "I want you to tell me how difficult it may be for you to do the following activities. The task may be:
1=NOT difficult, 2=MODERATELY difficult, 3=EXTREMELY difficult, or 4= IMPOSSIBLE.
9= Not be able to do the task for a reason not related to your vision loss.

Column B "I want you to think about how important it is for you is to be able to do this task without help from another person. You can answer:
1= Not important, 2=Moderately important 3=Very Important"

Column C "I want you to think about what items you might use to help you do this task.
1=own specs 2=other senses/ non-optical 3=someone helps me 4=wEVES 5=Optical LVA 6=EVES 7=Mainstream 9=cannot complete with any aids

Column D If someone else helps with the task, estimate how much help.
1=A lot, 2=50/50, 3=A little

Column E At Final wEVES VISIT ONLY ASK "Compared to next best solution wEVES are…"
1=Much worse, 2=Worse, 3=About the same, 4=Better, 5=Much better
You can also say 0 = Cannot Use wEVES for this task or 9=Only thing that I can use.

"These questions relate to Mobility activities. Remember, if you use a low vision device or adaptive technique to assist with the activity, please respond as though you were using the device or technique".					
	**A**	**B**	**C**	**D**	**E**
22. Get around outdoors in places you know					
23. Get around indoors in places you know					
24. Get around in unfamiliar places					
25. Go down steps in dim light					
26. Go out at night					
27. Get around in a crowd					
28. Avoid bumping into things					
29. Cross street at a traffic light					
30. Use public transportation					
31. Find public toilet					
32. Play sports					
33. Adjust to bright light					
34. Do gardening.					

A	4	Patient ID:				Date of Visit:					2	0		

Visual motor

Column A "I want you to tell me how difficult it may be for you to do the following activities. The task may be:
1=NOT difficult, 2=MODERATELY difficult, 3=EXTREMELY difficult, or 4= IMPOSSIBLE.
9= Not be able to do the task for a reason not related to your vision loss.

Column B "I want you to think about how important it is for you is to be able to do this task without help from another person. You can answer:
1= Not important, 2=Moderately important 3=Very Important"

Column C "I want you to think about what items you might use to help you do this task.
1=own specs 2=other senses/ non-optical 3=someone helps me 4=wEVES 5=Optical LVA 6=EVES 7=Mainstream 9=cannot complete with any aids

Column D If someone else helps with the task, estimate how much help.
1=A lot, 2=50/50, 3=A little

Column E At Final wEVES VISIT ONLY ASK "Compared to next best solution wEVES are…"
1=Much worse, 2=Worse, 3=About the same, 4=Better, 5=Much better
You can also say 0 = Cannot Use wEVES for this task or 9=Only thing that I can use

"These questions relate to Visual Motor activities. Remember, if you use a low vision device or adaptive technique to assist with the activity, please respond as though you were using the device or technique".

	A	B	C	D	E
35. Play table and card games					
36. Work on your favourite hobby					
37. Write a birthday card					
38. Sign your name					
39. Take a message					
40. Fix a snack					
41. Prepare meals					
42. Use appliance dials					
43. Groom yourself					
44. Eat and drink neatly					
45. Clean the house					
46. Physically get dressed					
47. Go to the cinema					
48. Go to spectator events					

A	5	Patient ID:				Date of Visit:					2	0		

Existing Coping Strategies and Use

Coping Strategies Listed in the first column.

Y/N = Y=participants has coping strategy N=participant does not have coping strategy

Column A

In the last 4 weeks how often have you used each device.

You can answer.

1=Never, 2=occasionally, 3= weekly, 4=1-4 daily, 5=>5 x day,

Column B

What is the longest in one go that you have used the device?

You can answer.

1= < 1 minute, 2=>1 minute and < 5 minutes, 3=>5 minutes and < 15 minutes, 4=>15 minutes and < 30 minutes, 5=>30minutes,

Coping Strategy	Y/N	A	B
Optical magnifiers near			
Optical Magnifiers distance			
EVES			
wEVES,			
Mainstream Technology as an LVA			
Non-optical			
Sensory Substitution			

A	6	Patient ID:				Date of Visit:					2	0		

Willingness to Pay: Ask at final wEVES visit.

"Your hand magnifier costs £50, an iPad costs £500, and a tabletop video magnifier costs approximately £2000. Imaging money was no problem; how much would you be willing to pay for this device?"

£						

1. **I found the Eye5 to be simple to understand.**
   - ☐ Strongly Agree
   - ☐ Agree
   - ☐ Neither Agree nor Disagree
   - ☐ Disagree
   - ☐ Strongly Disagree
2. **I found the Eye5 to be easy to use.**
   - ☐ Strongly Agree
   - ☐ Agree
   - ☐ Neither Agree nor Disagree
   - ☐ Disagree
   - ☐ Strongly Disagree
3. **If available, I would use the Eye5 in my daily life.**
   - ☐ Strongly Agree
   - ☐ Agree
   - ☐ Neither Agree nor Disagree
   - ☐ Disagree
   - ☐ Strongly Disagree
4. **What are the strengths of the device at present?**

5. **What are the weaknesses of the device at present?**
